# A Question about Out-Patients

**Published:** 1890-09-20

**Authors:** 


### Passing Topics.

### A QUESTION ABOUT OUT-PATIENTS.

A PARAGRAPH has been going the round of the papers to the effect that there are upwards of two hundred thousand separate families in London whose income is less than a pound a-week each. We do not know whence these figures are derived, or upon whose authority they are made public, but even though there were more reasons than are apparent for disputing the accuracy of the computation, and assuming that the estimate could be shown to be exaggerated, we think it would still afford a striking comment upon the efforts made in certain quarters to suppress the out-patients' departments of our hospitals. Whether or not these enormous aggregations of out-patients are desirable in the interests of science we will not now stop to inquire. Science will doubtless be able to take care of itself. What we would rather ask is, What bearing the figures quoted have upon the economic and philanthropic question?

We claim to be quite as sincere in our admiration of thrift and sturdy independence of character as the most rigid political economist or the strictest moralist, and it is not necessary at this time of day to admit that great reform is needed in the system of granting out-door treatment virtually to all comers. But without abating our reprobation of those persons who obtain charitable relief when not in need of it, or subtracting one iota from our appreciation of those qualities which, under some conditions, render even poverty independent of help, we may well ask what, when sickness supervenes, is to take the place of hospital relief in the case of families earning less than a pound a-week?

An average family may be reckoned to consist of five persons, and after payment of rent there would remain, of an income of £1, about three shillings a-piece to cover the cost of all other necessaries. To suppose that, although the bread-winner remains in good health, the means are sufficient to bear the cost of medical attendance would be nothing but a mockery of reason, while the alternative of the breadwinner's illness is not so infrequent that it may be left out of the reckoning.

It has often struck us as strange that a considerable number of well-intentioned and, as we have no cause to doubt, kindly, people are ready to impose upon the poorer classes a task which is practically an impossible one, even though the latter were endowed with a perfection of character and self-discipline not altogether human. For well-paid artisans and others who now too freely avail themselves of the benefits of our hospitals without making any return whatever, the proposals of the Provident system are admirable. The principles of the Metropolitan Provident Medical Association, as set forth upon page 4 of their report for 1889, are absolutely unimpeachable, but as the Association professedly deals only with "those members of the working classes and their families who are unable to pay the ordinary medical fees," we may fairly ask what of those who are unable to pay any fees at all? Surely a very large proportion of the 200,000 families must be in that position, and, moreover, many of those who might be provident are not, and never will be made so.

The real point therefore is, suppose the man to be so poor as to be unable, or so indifferent as to neglect to make provision for the time of future sickness, is charitable help to be denied him when the inevitable hour arrives? No doubt it is a duty much too often overlooked by the hospitals to guard against imposition, but it has never yet been contended that, given present sickness and poverty, they should refuse help because the patient was suspected of improvidence.

If the uncompromising critics of our hospitals know any way of avoiding the difficulties of the dilemma which presents itself, will they not grant us a plain exposition of the methods they have in view? We know the subject is an old one, but there is yet room for novelty in the treatment.

AGRICOLA.

